# Microbiota in Healthy Skin and in Atopic Eczema

**DOI:** 10.1155/2014/436921

**Published:** 2014-07-13

**Authors:** Giuseppe Baviera, Maria Chiara Leoni, Lucetta Capra, Francesca Cipriani, Giorgio Longo, Nunzia Maiello, Giampaolo Ricci, Elena Galli

**Affiliations:** ^1^National Health System Pediatrician ASL RMC-D6, Rome, Italy; ^2^Italian Society of Pediatric Allergy and Immunology (SIAIP), Atopic Dermatitis and Urticaria Committee, Italy; ^3^Pediatric Unit, Department of Clinical-Surgical, Diagnostic and Pediatric Sciences, University of Pavia, Pavia, Italy; ^4^Department of Reproduction and Pediatrics, University Hospital S. Anna, Ferrara, Italy; ^5^Pediatric Unit, Department of Medical and Surgical Sciences, University of Bologna, Bologna, Italy; ^6^University of Trieste, Trieste, Italy; ^7^Department of Woman, Child and General and Specialized Surgery, Second University of Naples, Naples, Italy; ^8^Pediatric Allergy Unit, Research Center, San Pietro Hospital-Fatebenefratelli, Rome, Italy

## Abstract

The Italian interest group (IG) on atopic eczema and urticaria is member of the Italian Society of Allergology and Immunology. The aim of our IG is to provide a platform for scientists, clinicians, and experts. In this review we discuss the role of skin microbiota not only in healthy skin but also in skin suffering from atopic dermatitis (AD). A Medline and Embase search was conducted for studies evaluating the role of skin microbiota. We examine microbiota composition and its development within days after birth; we describe the role of specific groups of microorganisms that colonize distinct anatomical niches and the biology and clinical relevance of antimicrobial peptides expressed in the skin. Specific AD disease states are characterized by concurrent and anticorrelated shifts in microbial diversity and proportion of *Staphylococcus*. These organisms may protect the host, defining them not as simple symbiotic microbes but rather as mutualistic microbes. These findings reveal links between microbial communities and inflammatory diseases such as AD and provide novel insights into global shifts of bacteria relevant to disease progression and treatment. This review also highlights recent observations on the importance of innate immune systems and the relationship with normal skin microflora for the maintenance of healthy skin.

## 1. Introduction

As it is constantly renewed, the epidermis sheds from the surface (desquamation) keratinocytes rich in microbes adhering to them [1] into the environment. It is the site of multiple exchanges between the body and the outside environment and represents a formidable physical barrier that protects the body from microbial attack from the outside environment and regulates loss of water and solutes [2]. The skin is not only an effective barrier between the organism and the environment, but also an ecosystem composed of different habitats rich in invaginations, pockets, and niches. Microorganisms inhabiting superficial skin layers are known as “skin microbiota” and include bacteria, viruses, archaea, and fungi.

In this review we focus on the role of skin microbiota not only in healthy skin but also in skin suffering from atopic dermatitis.

Every square centimeter of skin contains approximately 1 billion bacteria, including hair follicles and sebaceous glands [3]. These microbial communities are intimately involved in human welfare and disease [4–6]. In 2008 the National Institutes of Health launched the Human Microbiome Project with the aim of generating the resources and expertise needed to characterize the human microbiome and analyze its role in health and disease. It focused on studying the microbes residing in five body areas (skin, nose, mouth, stool, and vagina) in 250 “normal” adult volunteers [7]. Over 11,000 human specimens were obtained. Scientists then purified and sequenced the DNA from them and used information from the bacterially encoded 16S ribosomal RNA gene to identify and quantify the relative abundance of bacteria in each sample. In the microbial communities residing at different body sites four major groups, or phyla, were detected:* Firmicutes, Actinobacteria, Bacteroidetes*, and* Proteobacteria*. Specific groups of microorganisms colonize distinct anatomical niches: the most numerous microbes are well-defined resident flora. They are constantly present on body surfaces and may prevent colonization by pathogens and possible disease, restoring the ecological skin niches. Commensal microorganisms are in mutualistic symbiosis: they contribute to human health and welfare through the production of defense molecules or natural antibiotics. Transient skin flora can temporarily colonize the skin: these microorganisms are unable to remain in the body for a long period of time due to competition from resident microbes. They persist on the skin for a few hours or days and are not pathogenic under normal conditions (normal immune responses, skin barrier function intact).

## 2. Materials and Methods

A Medline and Embase search was conducted for studies evaluating the role of skin microbiota.

## 3. Results and Discussion

### 3.1. Development of Initial Human Skin Microbiota

The development of skin microbiota starts at birth. Sterile inside the uterus, the newborn is quickly colonized by microorganisms from the mother. In fact it has been proven that the fetus has already been in contact with microorganisms belonging to maternal microbiota. Meconium is the earliest stool of a newborn. Unlike later feces, meconium is composed of materials ingested during the time the infant spends in the sterile uterine environment: intestinal epithelial cells, lanugo, mucus, amniotic fluid, bile, and water.

Molecular studies define the diversity and abundance of microbes invading the amniotic cavity; studies in mice experimentally showed microbial placental exchange from mother to fetus [8–10]. The types of microbes found in meconium are influenced by maternal factors and may have consequences for the future health of the child. Gosalbes et al. [11] described different types of microbiota in meconium defined according to the taxonomic composition. In particular, the presence of enterobacteria was associated with a history of atopic eczema in the mother while the presence of lactobacilli was associated with respiratory disorders in the baby.

Delivery mode shapes the microbiota's establishment and, subsequently, its role in child health. Vaginally delivered infants acquired bacterial communities resembling their own mother**'**s vaginal microbiota and Cesarean-section infants harbored bacterial communities similar to those found on the skin surface [12].

The composition of cutaneous microbial communities evolves over the first year of life, showing increasing diversity with age. The infant skin microbiome is different from that of an adult, due to particular differences in skin structure and function. Although early colonization is dominated by* Staphylococci *(the stratum corneum of an infant is relatively better hydrated than that of an adult), their significant decline contributes to increased population evenness by the end of the first year. Similar to what has been shown in adults, the composition of infant skin microflora appears to be site specific. In contrast to adults, we find that* Firmicutes *predominate on infant skin. As the individual grows, the different microbial communities diversify, becoming similar to those of an adult organism by the age of 12–18 months [13].

### 3.2. Diversity of the Human Skin Microbiome

The skin surface varies topographically owing to regional differences in skin anatomy and, according to culture-based studies, these regions are known to support distinct sets of microorganisms. The density of sebaceous glands is a factor that influences the skin microbiota, depending on the region. Surface areas could be divided into dry, moist, or sebaceous environments regarding skin physiology and it has been demonstrated that these conditions are likely to influence the composition of the bacterial microbiome.

Eccrine sweat glands are the main sweat glands of the human body, found in virtually all skin. They produce a clear, odorless substance, consisting primarily of water and NaCl, which continuously wets the surface of the skin and produces a natural antibiotic, dermcidin. Areas with a high density of sebaceous glands, such as the face, chest, and back, encourage the growth of lipophilic microorganisms. In fact, sebaceous glands are connected near the top of hair follicles and produce the oily, waxy substance called sebum, which promotes the growth of facultative anaerobes such as* Propionibacterium acnes*, which, by hydrolyzing the triglycerides present in sebum, releases free fatty acids thereby contributing to the maintenance of the acidic epidermal pH [14]. Hair follicles and sebaceous glands represent an anoxic environment that hosts anaerobic microorganisms and produces cathelicidin LL37 and defensin (HBD-2).

Apocrine sweat glands are larger than eccrine sweat glands and are found only in the skin on certain areas of the body. These areas include the following: the underarms (axillae), under the breasts and around the nipples, and in the groin and genital region. They release their fluid (secretions) into the hair follicles, rather than directly on to the skin. The secretions are a thick, milky fluid, which can easily be turned into smelly body odour by germs (bacteria). It contains pheromones that respond to adrenaline and are related to sexual attractiveness.

16S ribosomal (r)RNA gene analysis is used as the standard for bacterial identification and bacterial taxonomic classification: the count of aerobic bacteria taken from areas such as the armpits or the folds between the toes can reach 10^7^ bacteria/cm^2^, while dry skin on the forearm or trunk may harbour 10^2^ bacteria/cm^2^. The colonization of bacteria is dependent on the physiology of the skin site, with specific bacteria being associated with moist, dry, and sebaceous microenvironments. The anaerobic bacterial colonization density of skin can reach 10^7^ CFU/cm^2^ [15]. The ecological body site niche is a greater determinant of the microbiota composition than individual genetic variation. The antecubital fossa, back, nare, and plantar heel are more similar to the same site on another individual than to any other site on the same individual.

Molecular analysis of the skin bacterial microbiota also revealed that its temporal variability depends on the body site [16, 17]. In healthy individuals the most consistent sites with respect to community membership and structure are the external auditory canal, inguinal crease, alar crease, and nare, whereas there is a significant variation on the second sampling of the popliteal fossa, volar forearm, and buttock, which suggests that longitudinal stability of the skin microbiome is site dependent. In general, contralateral sites on the same individual are more similar to each other than to a corresponding site on another individual [18]. Although there was a core set of bacterial taxa commonly found on the palm surface, there is a pronounced intra- and interpersonal variation in bacterial community composition: hands from the same individual shared only 17% of their phylotypes, with different individuals sharing a mere 13%. Moreover, bacterial population profile analysis showed that the bacterial communities on human hands were different according to the country considered [19]. Women had significantly higher diversity than men and community composition was significantly affected by handedness, time since last hand washing, and the individual's sex [20].

The composition of the skin microbiota affects the degree of attractiveness of human beings to the mosquito species (*Anopheles gambiae s.s.*). Microbial communities on the skin play key roles in the production of human body odour. Individuals that are highly attractive to* A. gambiae s.s.* have a significantly higher abundance, but lower diversity, of bacteria on their skin than individuals that are less attractive, and the volatile metabolites released by* Staphylococcus *spp. are attractive to* A. gambiae *females [21].

Recent work has demonstrated that the diversity of skin-associated bacterial communities is far higher than previously recognized, with a high degree of interindividual variability in the composition of bacterial communities. Given that skin bacterial communities are personalized, the authors hypothesized that the residual skin bacteria left on objects could be used for forensic identification, matching the bacteria on the object to the skin-associated bacteria of the individual who touched the object. Skin-associated bacteria can be readily recovered from surfaces (including individual computer keys and computer mice) and the structure of these communities can be used to differentiate objects handled by different individuals, even if those objects have been left untouched for up to 2 weeks at room temperature [22].

### 3.3. The Role of* Staphylococcus epidermidis*

The multilayered structure of skin reflects the complexity of its multifunctional activities. The skin is a physical and chemical barrier between the outside environment and the tissues inside the body; the skin is one of several organ systems participating to the maintenance of a core temperature; the skin acquires sensory information from the environment and relies on innate defense mechanisms inducing the release of antimicrobial peptides (AMPs) such as cathelicidin LL37 or beta-defensins. These antimicrobial peptides, which are synthesized in the skin at sites of potential microbial entry, provide a soluble barrier that acts as an impediment to infection. Recent studies have revealed that our skin's innate immune system is not solely of human origin. The commensal microbes themselves produce antimicrobial peptides (AMPs) able to increase the production of AMPs by keratinocytes. Thus the skin helps to maintain homeostasis by suppressing excess cytokine release after minor epidermal injury [23]. The unique peptides, phenol-soluble modulin (PSM)*γ* and PSM*δ* produced by* Staphylococcus epidermidis *(*S. epidermidis*), could be beneficial to the host and thus serve as additional AMPs on normal skin surface. These peptides possess two opposite sides organized by their hydrophobic and cationic amino acids with a five-amino acid periodicity, a strategy for the action of both a hydrophilic and hydrophobic molecule that resembles that of classic AMPs such as LL-37. These peptides selectively exhibited bactericidal activity against skin pathogens, such as* Staphylococcus aureus *(*S. aureus*), Group A* Streptococcus *(GAS), and* Escherichia coli*, whereas they are not active against* S. epidermidis*. This selective activity is likely to be an important part of a normal microbial defense strategy against colonization and in maintaining the normal microbial ecosystem [24].

*Staphylococcus epidermidis *is a Gram-positive bacterium and it comprises more than 90% of the aerobic resident flora. Recent studies can be interpreted to suggest that* S. epidermidis *is a mutualistic organism, much like the bacteria in the gut [25]: the bacteria primarily infect compromised patients. Many strains of* S. epidermidis *produce lantibiotics, which are lanthionine containing antibacterial peptides, also known as bacteriocins. The several identified bacteriocins include epidermin, epilancin K7, epilancin 15X, Pep5, and staphylococcin 1580 [26–28]. The host epidermis permits* S. epidermidis *growth as the bacterium may provide an added level of protection against certain common pathogens, making the host-bacterium relationship one of mutualism. Many strains of* S. epidermidis *produce antibacterial peptides (<10 KE) that amplify the keratinocyte response to pathogens via TLR2. In fact,* S. epidermidis *plays an additional protective role by influencing the innate immune response of keratinocytes through Toll-like receptors (TLRs) signaling maintenance of the skin barrier function and integrity. Activation of TLR-2 by* S. epidermidis* enhances the expression of tight junctions and decreases the production of proinflammatory cytokines via TLR3 in the keratinocyte cultures [29, 30]. Through cell “priming,” keratinocytes are able to respond more effectively and efficiently to pathogenic insults [31].

Biological control might be a new possible way of controlling* Staphylococcus aureus *in body surfaces. Colonization of body surfaces (especially in the nose) by* S. epidermidis *impairs the establishment of* S. aureus*. It was discovered that there are two different strains of* S. epidermidis*, one that inhibits biofilm formation by* S. aureus*,* S. epidermidis *strain JK16 (inhibitory type), and one that does not (noninhibitory type),* S. epidermidis *strain JK11 [32, 33]. In vivo studies have shown that Esp-secreting* S. epidermidis *eliminates* S. aureus *nasal colonization [34]. In fact, the serine protease Esp [35–37] secreted by a subset of* S. epidermidis*, a commensal bacterium, inhibits biofilm formation and nasal colonization by* S. aureus*, a human pathogen. Epidemiological studies have demonstrated that the presence of Esp-secreting* S. epidermidis *in the nasal cavities of human volunteers correlates with the absence of* S. aureus*. Purified Esp inhibits biofilm formation and destroys preexisting* S. aureus *biofilms. Furthermore, Esp enhances the susceptibility of* S. aureus *in biofilms to immune system components through human beta-defensin-2 (hBD2). This is due to an amensalistic relationship between these microorganisms, the inhibitory strain of* S. epidermidis *and* S. aureus *[38].

### 3.4. *Staphylococcus aureus* and Atopic Eczema

The incidence of atopic eczema has risen over the last few decades, now affecting 15–20% of the infant population. 20–40% have an innate genetic filaggrin mutation: FLG mutations simply confer a risk for allergen sensitization through the skin, leading to increased transepidermal water loss (TEWL), including increased surface pH and altered expression of antimicrobial peptides.* S. aureus *is nonmotile, nonspore forming, and catalase and coagulase positive. Typical colonies are yellow to golden yellow in color, smooth, entire, slightly raised, and hemolytic on 5% sheep blood agar.* S. aureus *is extremely prevalent in people with atopic dermatitis.* S. aureus *is predominantly localized in the anterior nares (*vestibulum nasi*): ~20% of individuals are persistent* S. aureus *carriers, ~60% are intermittent carriers, and 20% are persistent noncarriers [39, 40].

The biology of nasal colonization with* S. aureus *is not fully understood. A variety of bacterial factors have been deemed important for the maintenance of colonization of the human nasal cavity by* S. aureus*. In addition, environmental factors, as well as host factors of the immune status, are thought to play a pivotal role in determining the* S. aureus *nasal carrier state. Several glucocorticoid receptor (GR) gene polymorphisms are thought to be functional and have been described as associated also with variation in glucocorticoid sensitivity. Consequently, changes in glucocorticoid sensitivity may predispose to or protect from microbial colonization or infection on the one hand or autoimmune disease on the other. For example, homozygous presence of haplotype 3 conferred a 68% lower risk of persistent* S. aureus *nasal carriage. Carriers of haplotype 5 were at an increased risk of persistent* S. aureus *carriage. People with the genotypic combination of haplotype 1 and this haplotype allele had an 80% higher risk of persistent* S. aureus *carriage than all other genotypes [41]. Syed et al. demonstrated a novel environmental factor that can influence the ability of* S. aureus* to bind to surfaces altering* S. aureus* nasal colonization. In fact, they showed that the biocide triclosan is commonly found in the nasal secretions of healthy adults and the presence of triclosan has a positive trend with nasal colonization by* S. aureus* [42].

In order to study how* S. aureus *persists in the skin over time and what bacterial factors it may use to actively modify the host skin environment to persist in its replicative niche, Popov et al. proposed a three-dimensional (3D) human skin culture model as an informative and tractable experimental system for future investigations of the interactions between* S. aureus *and multifaceted skin tissue. Studies using a 3D organotypic human epidermal tissue model could examine how* S. aureus *and immune cells interact with one another in stratified human epidermal tissue and assess how immunization against* S. aureus* might alter bacterial population behavior on the epidermis or protect against invasive epidermal infections [43].

Why are over 90% of AD patients colonized with* S. aureus*? In normal skin, microorganisms are recognized by innate immune receptors such as Toll-like receptors on the keratinocyte surface and keratinocytes produce antimicrobial molecules such as HBD-2, HBD-3, and NO. IL-8 (neutrophil chemokine) is also produced to drive neutrophils from the bone marrow pool into inflamed skin. In a Th2 environment, IL-4 and IL-13 induce the phosphorylation of STAT6 which inhibits INF-*γ* and TNF-*α*. This in turn inhibits the production of HBD-2 and HBD-3, causes a reduction in the production of IL-8, and results in defective neutrophil accumulation in the skin. These events may all contribute to allowing microbes to grow in the Th2 environment of AD skin [44].

## 4. Conclusions

The skin is an active immune organ in which the keratinocytes can no longer be considered as cells that have now died and have the only function of acting as a barrier against the external environment; rather, they must be considered as active components of the immunoregulatory network between the external environment, the resident cutaneous immune system, and the microbiota [45].

Bacterial stimuli cause the production of antimicrobial peptides (AMPs) and proinflammatory cytokines [46] which interact with memory T-cells resident in the skin itself. The interaction with the cutaneous bacterial flora is essential, for example, in order to promote effective T-cell response against infections from* L. major* [47] while the colonization of the skin of people with atopic dermatitis by* Staphylococcus aureus* activates local inflammatory processes through the release of *∂*-toxin [48].

Atopic dermatitis is a chronic, relapsing, and intensely pruritic inflammatory skin disorder; specific AD disease states are characterized by concurrent and anticorrelated shifts in microbial diversity and proportion of* Staphylococcus*. There is a strong association between worsening disease severity and lower skin bacterial diversity. AD flares are characterized by low bacterial diversity in the absence of recent treatment. In contrast, intermittent or active treatment is associated with higher bacterial diversity.* S. aureus *is observed during disease flares; the use of AD treatments modifies microbial diversity and proportions of* Staphylococcus*. Antimicrobial or anti-inflammatory medications decreased* S. aureus *predominance, affecting bacterial diversity during flares. Consistent and continued treatment over a period of time is required to induce the* resolving flare* stage, which transides into a restoration of full microbial diversity and low population levels of* Staphylococcus*, typical of a true postflare. Increases in the proportion of* Staphylococcus *and reductions in microbial diversity precede the worsening of AD disease severity as observed in no-treatment flares [49].

*Meta*“*omics*”* studies* are on the verge of revolutionizing our perspective on skin bacterial flora, the factors that determine which microorganisms colonize the skin, their relationship with the innate and adaptive immune system, and the possibility to use microbiota or probiotics as therapeutic agents. These are all hypotheses that open up to future research and for which further studies are required in order to reveal the mysterious interactions of an infinitely small highly populated world and the organism that hosts it [50, 51].
